# Prevalence of Smoking among Men in Ethiopia and Kenya: A Cross-Sectional Study

**DOI:** 10.3390/ijerph15061232

**Published:** 2018-06-11

**Authors:** Shangfeng Tang, Ghose Bishwajit, Tegene Regassa Luba, Sanni Yaya

**Affiliations:** 1 School of Medicine and Health Management, Tongji Medical College, Huazhong University of Science and Technology, Wuhan 430030, China; sftang@hust.edu.cn (S.T.); elias.tegene@yahoo.com (T.R.L.); 2 Faculty of Social Sciences, School of International Development and Global Studies, University of Ottawa, Ottawa, ON K1N 6N5, Canada; Sanni.yaya@uottawa.ca; 3 Federal Ministry of Health, Addis Ababa 8744, Ethiopia

**Keywords:** Demographic and Health Survey, Ethiopia, Kenya, smoking, tobacco control

## Abstract

While tobacco use remains the largest single cause of premature death in the industrialized countries, low-and-middle income countries are also experiencing a rising burden of the tobacco epidemic and are making various programmatic efforts to tackle the issue. Evidence-based policy making is critical to the long-term success of tobacco intervention programs and is reliant on regular monitoring of the trends and prevalence rates of tobacco use though population-based surveys, which are sparse for countries in eastern Africa. Therefore, in the present study we aimed to (1) estimate the trends in the prevalence of self-reported smoking status; and (2) explore the sociodemographic factors associated with smoking among adult men in Ethiopia and Kenya. *Methods*: Subjects were 26,919 adult men aged between 15 and 59 years from Ethiopia and Kenya. Nationally representative cross-sectional data on self-reported smoking and relevant sociodemographic indicators were collected from the Demographic and Health Surveys (DHS) conducted in these two countries. Data analysis was performed by descriptive, bivariate, and multivariable methods. *Results*: In Ethiopia, the prevalence rate of smoking increased from 8.5% in 2005 to 11.7% in 2011. While in Kenya, the rate declined albeit slowly from 22.9% in 2003 to 18.8% in 2008–2009 and 17% in 2014. The prevalence was significantly different in urban and rural areas. In majority of the surveys, prevalence of smoking was highest in the age group of 25–34 years. The prevalence of smoking varied widely across several socioeconomic characteristics. *Conclusions*: The findings indicate a high rate of smoking among men especially in urban areas, and call for policy actions to address the socioeconomic factors as a part of the policy to strengthen tobacco-control efforts.

## 1. Introduction

Smoking-attributable mortality (SAM) is a serious public health concern in the 21st century which now outnumbers that from human immunodeficiency virus (HIV), tuberculosis, and malaria combined [1]. According to the 2013 World Health Organization (WHO) report on the global tobacco epidemic, tobacco use accounts for millions of premature deaths and billions of dollars of economic damage every year [2]. Apart from the health issues, tobacco use has significant bearings on socioeconomic status, quality of life and general well-being. Some of the common impacts of tobacco use include direct expenses to purchase tobacco products, medical expenditure for treating tobacco-related illnesses and lower workplace productivity, which can contribute substantially to household poverty, poor nutrition and low education, particularly in low- and middle-income countries (LMICs) [3]. Many LMICs are undergoing early stages of the tobacco epidemic with a lower burden of tobacco use and SAM compared with the high-income countries (HICs): 18% in the HICs vs. 11% in middle-income countries and 4% in LMICs [4]. However, the prevalence of smoking in the LMICs is increasing at a fast pace which is projected to account for a higher proportion of SAM during the coming decades. Between 1990 and 2009, the prevalence of tobacco use in the Western Europe declined by a quarter (26%), while that in Africa and some Middle Eastern countries rose by more than half during the same period (57%) [5].

The slow decline of tobacco use in developed countries has resulted in a focus-shift of the tobacco industry from the West to emerging economies in Africa and Asia [6,7]. During 2003–2012, the total area harvested for tobacco in Africa increased by two-thirds, while in the USA and Europe that decreased by 18% and 40.4%, respectively [7]. In the context of African countries, this is particularly worrisome as the underdeveloped healthcare systems struggle to cope with the rising prevalence of non-communicable chronic diseases (NCDs) [6]. In the wake of this situation, there has been growing concern regarding tobacco control in African countries in recent years as more and more countries are willing to implement the WHO Framework Convention on Tobacco Control (FCTC) [8,9]. Kenya, as the largest economy in eastern Africa, has shown concrete commitments for tobacco control policies and has become a signatory of the FCTC framework since 2004. Ethiopia, which is projected to surpass Kenya to become the largest economy in the region, has ratified the framework convention in 2014. 

As two fast-growing economies, both Ethiopia and Kenya have been experiencing an expanding middle class with ensuing alternations in demographic, dietary and epidemiological structures marked by the rising prevalence of lifestyle diseases including tobacco-induced ones [10,11]. Hence, reducing the use of tobacco at the population level remains a key imperative from both healthcare and national development aspects. A major requirement for developing and achieving tobacco control targets is the availability of workable information on the magnitude of the burden and underlying causes. However, research evidence on the nationwide prevalence of tobacco use is scarce in Ethiopia and Kenya. Previous research on smoking in these two countries was either based on non-representative samples at sub-national level, or failed to show the trends in prevalence rates [12,13]. As the case with most low-income settings, resources for comprehensive surveys are limited in Ethiopia and Kenya. For this purpose, in the present study we used datasets from the Demographic and Health Survey (DHS) which provide high-quality data on various indicators of population health and risk factors such as tobacco smoking and alcohol drinking. Based on these datasets, the aim of this study was to estimate the trends in prevalence of smoking among adult men aged 15–59 years. In addition, we also investigated the association of smoking with several sociodemographic and economic variables. 

## 2. Methods

### 2.1. Data Collection

Datasets used in this study were collected from the Demographic and Health Surveys (DHS) conducted since 2000 in Ethiopia and Kenya. DHS surveys are country-representative cross-sectional surveys providing quality information on a wide range of health, illness, and healthcare utilization and behavior-related indicators with the aim of enabling the host countries to develop evidence-based health policies and monitor the progress of health programs. These surveys are part of the worldwide MEASURE DHS project funded by the United States Agency for International Development (USAID) with technical assistance from ICF (Inner City Fund) International [14]. In Ethiopia, the surveys are implemented by the Ethiopian Central Statistical Agency (CSA), and by Kenya National Bureau of Statistic in Kenya. Data from the fifth (2005) and sixth (2011) round of the survey in Ethiopia, and fourth (2003), fifth (2008–2009) and seventh (2014) round in Kenya (data for sixth round was not available) were analysed. Survey and sampling methods were published by DHS and are available in the final reports [14,15].

### 2.2. Study Settings

Geographically, both Ethiopia and Kenya are situated in eastern Africa sharing a border of 867 km. Ethiopia is administratively divided into 11 regions (Nine regional states and two city administrations) covering about 1.1 million km^2^, while with its 47 administrative counties Kenya occupies an area of 582,646 km^2^. Each of the 47 counties are stratified into urban and rural strata producing a total of 92 strata for the survey sampling (Nairobi and Mombasa have only urban areas). In Ethiopia, each administrative region is divided into zones, zones into *weredas* (smaller administrative units), and *weredas* into *kebeles* (smallest administrative units). In the surveys, each *kebele* is further divided into enumeration areas (EAs) for convenient implementation of the census. In the 2011 EDHS, sample area included a total of 624 EAs with 187 in urban areas and the rest from the rural areas. 

### 2.3. Study Variables

The outcome variable in the study was self-reported smoking status. Participants were asked whether they currently smoke cigarettes and, if so, how many cigarettes they had smoked in the past 24 h. Based on literature review, various demographic and socioeconomic variables were included in the analysis and were categorized as—Age: 15–24, 25–34, 35–44, 45+; Marital status: Single, Married, Widow/divorced; Religion: Christian, Moslem, Other; Education: Nil, Primary, Secondary, Higher; Occupation: Agriculture, Managerial, Sales/service, Manual, Unemployed; Wealth status: Poorest, Poorer, Middle, Richer, Richest; Drinks alcohol: No, Yes [6,7,8,10,12,16,17,18,19]. 

### 2.4. Data Analysis

Datasets were checked for missing values, outliers and were weighed prior to analysis. Prevalence rates of smoking were presented as percentages. Secondly, cross-tabulation was performed with chi-square tests of association to check which sociodemographic variables were associated with smoking. The analysis was stratified by region as people in urban and rural areas tend to vary substantially in terms of health-related behavior. The variables that were significant at *p* < 0.25 were selected for multivariable regression analysis. Two separate models were run for each country. Results of regression analysis were presented as odds ratios and 95% confidence intervals (CIs). Statistical significance was set at *p* < 0.05 (two-sided) for the final regression analysis. All analyses were carried out using SPSS software version 24. 

### 2.5. Ethics Approval

Participants gave informed consent before taking part in the survey. All DHS surveys are approved by ICF International as well as an institutional review board (IRB) in the host country to make sure that the protocols are in compliance with the U.S. Department of Health and Human Services regulations for the protection of human subjects.

## 3. Results

### 3.1. Prevalence of Smoking

Table 1 shows that the prevalence of smoking in Ethiopia increased from 8.5% in 2005 to 11.7% in 2011. In Kenya on the other hand, the prevalence decreased gradually from 22.9% in 2003 to 18.8% in 2008–2009 and 17% in 2014 (Table 2). Except for in Ethiopia in 2005, urban men had a significantly higher prevalence of smoking compared with their rural counterparts, and the rate was highest among men aged between 25–34 years.

### 3.2. Participant Characteristics

Basic sample characteristics were presented in Table 3. In both countries, a majority of the men were of rural origin. The mean age of men in Ethiopia and Kenya were, respectively, 30.59 (standard deviation (SD) 11.57) and 30.08 (SD 10.93). In both Ethiopia and Kenya, more than one-third of the participants were aged between 15 to 24 years which indicated that the sample population was predominantly young. More than half of the men were unmarried (53.4% in Ethiopia and 50.2% in Kenya), and belonged to the Christian faith (60.1% in Ethiopia and 83.6% in Kenya). The rate of literacy was higher Kenya (94% vs. 68.5%) and majority of the participants had a primary level of qualification (47.3% in Ethiopia and 51.1% in Kenya). Agriculture was the most common profession among both urban and rural men in both countries. The rate of unemployment was respectively 9.4% and 17.9%. Regarding household wealth status, overall about one-fifth of the men were living in the poorest households with the percentage being noticeably higher in rural areas compared to urban areas (2.1% vs. 27.9% in Ethiopia and 9.2% vs. 28.2% in Kenya). About half (51.2%) of the men in Ethiopia and over a quarter (26.8%) in Kenya reported drinking alcohol. The prevalence of drinking was higher among urban men in both countries. 

### 3.3. Cross-Tabulation

Results of cross-tabulation showing the bivariate association between smoking and the sociodemographic characteristics are presented in Table 4. The results indicate that the prevalence of smoking was higher among men who were aged 25–34 years, unmarried, Christian (Kenya) or Moslem (Ethiopia), had primary-level education, and were employed in agriculture, living in the poorest households (except for urban men in Ethiopia), and drank alcohol. 

### 3.4. Multivariable Analysis

Results of multivariable analysis on the association between smoking and sociodemographic variables are presented in Table 5. The results indicate that in Kenya, the likelihood of being a smoker increased with age, and the odds of smoking was highest in the age group of 25–34 years. Compared to those in the 15–24 age group, the odds of smoking were 4.2 (odds ratio (OR) = 4.155, 95% CI = 3.026–5.704) and 3.8 times (OR = 3.795, 95%CI = 2.930–4.916) higher among men aged 25–34 years in urban and rural areas respectively. Among those who belonged to Moslem faith, the odds of smoking was 71% higher among men in rural Ethiopia, but 63% and 50% lower in urban and rural Kenya compared to those who belonged to the Christian faith. Compared to those who had no education, the odds of smoking was 42% higher (OR = 1.421, 95%CI = 1.086–1.859) in urban Ethiopia, and, respectively, 55% (OR = 1.547, 95%CI = 1.087–2.697) and 50% (OR = 1.504, 95%CI = 1.086–2.081) higher in urban and rural Kenya. In Ethiopia, those who were employed in sales/service and manual jobs had, respectively, 70% (OR = 1.7, 95%CI = 1.164–2.483) and 79% (OR = 1.788, 95%CI = 1.262–2.534), while in Kenya, employment in managerial and sales/service jobs were associated with, respectively, 36% (OR = 0.643, 95%CI = 0.485–0.854) and 30% (OR = 0.702, 95%CI = 0.496–0.996) lower odds of smoking. Living in the richest households was associated with higher odds of smoking in Ethiopia (OR = 1.624, 95%CI = 1.093–2.413), but lower in Kenya (OR = 0.507, 95%CI = 0.389–0.662). Alcohol-drinking showed significantly higher association with smoking in Kenya (5.8 and 6.4 times higher odds in urban and rural areas), but not in Ethiopia. 

## 4. Discussion

Effective policy-making for controlling tobacco use requires routine monitoring of prevalence rates. With that purpose in mind, in the present study we have analyzed the Demographic and Health Survey datasets conducted in last 15 years in Ethiopia and Kenya and have shown the trends and current prevalence of smoking among adult men. Findings indicated that in Ethiopia the prevalence of smoking has increased since the 2005 survey, while in Kenya the rate has been decreasing at a slow pace since the 2003 survey. The declining trend in Kenya might be due to the implementation of the Framework Convention on Tobacco Control of the WHO (which has been in effect since 2005) to which the country was one of the earliest signatories [17]. Ethiopia, on the other hand, has joined the treaty only recently (2014); however the overall prevalence in Ethiopia was still lower than that of Kenya (17% in 2014 in Kenya vs. 11.7% in 2011 in Ethiopia), and of the African Region average of 15.8% (2010 estimate) [18]. Data were not available for subjects under 15 year of age, however the Global Youth Tobacco Survey of 2007 reported an equally high prevalence of tobacco consumption (18.5%) among youth aged 13 to 15 years. Analysis of the data on 23 African countries from the Global Progress Report on implementation of the treaty reveals that the rate of implementation varied substantially, ranging from 9% in Sierra Leone to 78% in Kenya [19]. Thus, the high rate of smoking in Kenya comes as a surprise given the highest rate of implementation of the framework. 

Findings also indicated the existence of regional disparities in the prevalence of smoking as urban men were significantly more likely to report smoking, except for the 2005 DHS survey in Ethiopia. Although the population in Eastern African region is predominantly rural, both countries are urbanizing at a fast pace led by the recent boom in employment in urban areas, which is usually associated with higher adoption of smoking behavior [20]. As urban and rural people share varying degrees of exposure to environmental and lifestyle-related risk factors, understanding the geographical variation in smoking patterns is essential for regional priority setting and policy actions. The findings of this study support the fact that urban men should be regarded as a priority concern for smoking interventions, especially the age group of 25 to 34 years. 

Religious affiliation also appeared to be significantly associated with smoking behavior among both urban and rural men in Kenya. In the context of the present study, it is hard to discern the causality of this relationship; however, previous studies have reported that religious commitment is an important predictor of lifestyle and health-related behavior [21,22]. Given the considerably high percentage of religious attachment in this region, this can be regarded as an opportunity for the promotion of healthier lifestyles through various religious teachings and relevant norm-setting that discourages smoking and other unhealthy behavior which may lead to greater health outcomes and social well-being [23]. 

With regard to education, the association was significant in the urban areas only and was relatively stronger for Kenya than in Ethiopia. Surprisingly, compared to men who had no formal education, the likelihood of being a smoker was higher among those with a higher educational profile. While the finding appears to be counterintuitive, this is in line some previous studies [24] and inconsistent with others [25,26]. In general, higher educational attainment is associated with better self-efficacy and adherence to healthy behavior. However, the relationship may not be monotonic and subject to variation depending on sociocultural and environmental factors such as the level of exposure to smoking advertisements, stages of urbanization, and degrees to which anti-smoking policies are applied. As countries tend to urbanize, an increasing proportion of the labor force shifts from agriculture/manual to service/managerial jobs indicating a higher socioeconomic status and disposable income, which can serve as an enabling factor for healthier lifestyles. Findings from Kenya also support these facts as men employed in service and managerial jobs in rural areas and with higher wealth status in the urban areas had significantly lower odds of smoking. However, interpretation of these findings should be considered in the context of the relevant factors (e.g., aggressive marketing, social smoking, occupational prestige) deterring abstinence from or cessation of smoking. 

Previous studies from developed countries have shown that higher income and educational status can influence increased consumption of alcohol [27], which can trigger higher consumption of tobacco [28]. Our findings suggest that alcohol consumption was associated with significantly higher odds of smoking among both urban and rural men in Kenya. This finding suggests that anti-smoking policies and campaigns should also focus on addressing alcohol use/abuse to effectively reduce the prevalence of smoking.

Although our findings do not indicate any causal relationship between smoking and demographic and socioeconomic variables, some interesting contrasts emerged from the analysis. As the associations tended to be more significant for Kenya than in Ethiopia, this might indicate dissimilarities in the underlying determinants of smoking in these countries. Further comparative studies will be required by including more context-specific variables associated with the adoption of smoking, success in cessation, and effectiveness of the current tobacco-control programs. Clearly, both of the counties are far from achieving their full potential in terms of anti-smoking policy development and implementation of the Framework Convention on Tobacco Control program. One important policy implication of the findings of the present study is that tobacco-control programs need to take into account the age and regional differentials along with the socioeconomic factors relevant to the standard of living among smokers (e.g., education, economic status). Long-term success in reducing the burden of smoking will require appropriate social policy instrumentation to address social factors, as well as political commitment against tobacco production and marketing that can undermine the efficacy of the programmatic efforts. 

As far as we are concerned, this is the first study to estimate the trend in the prevalence of smoking among adult men in eastern Africa. Sample size was relatively large and representative of men aged between 15 and 59 years. The findings also provide important insights for policy action and for more comprehensive studies in both countries. However, besides the important contributions our study makes, it has several limitations that need attention when interpreting the results. Firstly, as the data were secondary, this limited the choice and measurement of the variables. As such, some key variables relevant to tobacco-smoking behaviour were not possible to include in the study. The prevalence of smoking and other variables was self-reported, which is subject to reporting bias. The results represent the prevalence of smoking only, and not the overall prevalence, as other forms of tobacco use were not considered. Also, the associations derived from the cross-sectional analysis cannot confirm any causality. 

## 5. Conclusions

In conclusion, the findings indicate that the prevalence of smoking has increased in Ethiopia since the last survey conducted in 2005. Kenya, on the other hand, has been experiencing a slow reduction in the prevalence rate over the course of the last decade. Important differences in the prevalence were observed across age groups and regions. Interestingly, although Kenya had adopted the FCTC framework a decade earlier than Ethiopia, the prevalence of smoking appeared to be higher than in Ethiopia nonetheless. Results suggest that tobacco-control programs need to emphasize addressing the demographic and socioeconomic determinants that influence smoking among men in the region. Based on the insights from the current literature, it is suggested that both of the countries continue to strengthen their commitments to tobacco control and intensify policy actions against the marketing and promotion efforts of Western tobacco companies.

## Figures and Tables

**Table 1:** Trends in the prevalence (%) of smoking among men aged 15 years and above in Ethiopia. Ethiopia Demographic and Health Survey.

	2005				2011			
Age	Total (8.5)	Urban (8.1)	Rural (8.6)	*p*	Total (11.7)	Urban (13.4)	Rural (11)	*p*
15–24	10.9	10.8	11.1	0.000	15.9	15.9	15.9	0.000
25–34	27.9	33.8	26.8		34.8	37.3	33.4	
35–44	38.0	37.8	38.0		28.9	29.2	28.7	
45+	23.2	17.6	24.1		20.4	17.6	21.9	

**Table 2:** Trends in the prevalence (%) of smoking among men aged 15 years and above in Kenya. Kenya Demographic and Health Survey.

	2003				2008–2009				2014			
Age	Total (22.9)	Urban (23.5)	Rural (22.7)	*p*	Total (18.8)	Urban (19.6)	Rural (18.4)	*p*	Total (17)	Urban (17.3)	Rural (16.8)	*p*
15–24	23.1	23.8	22.8	0.000	18.8	18.9	18.7	0.000	11.1	12.5	10.3	0.000
25–34	34.4	41.6	32.0		36.5	39.6	34.9		36.0	39.1	34.1	
35–44	29.2	24.8	30.7		26.3	27.8	25.6		30.9	28.8	32.3	
45+	13.3	9.8	14.5		18.5	13.7	20.8		21.9	19.6	23.3	

**Table 3:** Sociodemographic characteristics of the sample population.

	Ethiopia			Kenya		
	Total (14,104)	Urban (29.9)	Rural (70.1)	Total (12,815)	Urban (38.3)	Rural (61.7)
Age						
15–24	5160 (36.6)	37.0	36.4	4792 (37.4)	33.7	39.7
25–34	3955 (28.0)	30.9	26.8	3641 (28.4)	33.1	25.5
35–44	2787 (19.8)	19.2	20.0	2684 (20.9)	21.4	20.7
45+	2202 (15.6)	12.8	18.8	1698 (13.3)	11.8	14.1
Marital status						
Single	7536 (53.4)	54.3	53.1	6437 (50.2)	49.0	51.0
Married	6177 (43.8)	43.2	44.1	6124 (47.8)	47.4	48.0
Widow/divorced	391 (2.8)	2.5	2.9	254 (2)	3.6	1.0
Religion						
Christian	8478 (60.1)	68.0	56.7	10709 (83.6)	81.6	84.8
Moslem	5311 (37.7)	31.4	40.3	1564 (12.2)	15.2	10.4
Other	315 (2.2)	0.6	2.9	542 (4.2)	3.2	4.9
Education						
Nil	4445 (31.5)	7.8	41.6	766 (6.0)	3.4	7.6
Primary	6669 (47.3)	39.7	50.5	6548 (51.1)	42.0	56.8
Secondary	1626 (11.5)	26.8	5.0	4062 (31.7)	36.6	28.6
Higher	1364 (9.7)	25.6	2.9	1439 (11.2)	18.0	7.0
Occupation						
Agriculture	8464 (60.0)	57.2	61.2	5135 (40.1)	30.2	46.2
Managerial	1086 (7.7)	8.3	7.5	1533 (12.0)	17.2	8.7
Sales/service	1747 (12.4)	13.6	11.9	655 (5.1)	7.3	3.7
Manual	1487 (10.5)	11.1	10.3	3198 (25.0)	28.7	22.6
Unemployed	1320 (9.4)	9.8	9.2	2294 (17.9)	16.5	18.8
Wealth status						
Poorest	2846 (20.2)	2.1	27.9	2682 (20.9)	9.2	28.2
Poorer	2108 (14.9)	0.6	21.1	2577 (20.1)	12.3	25.0
Middle	2152 (15.3)	0.5	21.5	2636 (20.6)	13.9	24.7
Richer	2403 (17.0)	4.2	22.5	2758 (21.5)	28.8	17.0
Richest	4595 (32.6)	92.6	7.0	2162 (16.9)	35.8	5.1
Drinks alcohol						
No	6880 (48.8)	47.9	49.2	9376 (73.2)	70.4	74.9
Yes	7224 (51.2)	52.1	50.8	3439 (26.8)	29.6	25.1

**Table 4:** Prevalence of smoking across the sociodemographic characteristics among men in Ethiopia and Kenya.

	Ethiopia		Kenya	
	Urban	Rural	Urban	Rural
Age				
15–24	15.9	15.9	12.5	10.3
25–34	37.3	33.4	39.1	34.1
35–44	29.2	28.7	28.8	32.3
45+	17.6	21.9	19.6	23.3
*p*-Value	0.000	0.000	0.000	0.000
Marital status				
Single	55.4	51.8	50.2	49.7
Married	42.7	45.5	45.9	49.6
Widow/divorced	1.9	2.7	3.9	0.7
*p*-Value	0.598	0.55	0.577	0.208
Religion				
Christian	49.2	24.2	77.2	82.8
Moslem	50.1	72.0	16.5	9.1
Other	0.7	3.8	6.4	8.2
*p*-Value	0.000	0.000	0.000	0.000
Education				
Nil	12.9	49.0	14.0	16.0
Primary	40.0	45.1	46.8	49.6
Secondary	27.1	3.9	29.6	19.5
Higher	20.0	2.0	9.5	14.8
*p*-value	0.000	0.000	0.000	0.000
Occupation				
Agriculture	59.5	57.8	18.8	23.9
Managerial	8.8	9.1	17.2	28.0
Sales/service	13.6	15.2	11.4	29.7
Manual	10.1	10.4	27.5	14.0
Unemployed	8.0	7.6	25.1	4.4
*p*-Value	0.449	0.000	0.000	0.002
Wealth status				
Poorest	2.1	36.6	40.1	59.8
Poorer	0.5	19.1	11.9	6.6
Middle	0.7	16.4	7.8	3.6
Richer	6.9	19.8	37.4	28.2
Richest	89.7	8.0	2.8	1.8
*p*-Value	0.017	0.000	0.000	0.000
Drinks alcohol				
No	47.4	47.5	37.8	35.4
Yes	52.6	52.5	62.2	64.6
*p*-Value	0.821	0.274	0.000	0.000

N.B. *p*-value calculated from chi-square tests.

**Table 5:** Association between smoking and sociodemographic variables.

	Ethiopia * (Odds Ratio (OR), 95%CI)		Kenya ** (OR, 95%CI)	
	Urban	Rural	Urban	Rural
Age				
15–24	ref	ref	ref	ref
25–34	0.280 (0.160–0.490)	0.274 (0.191–0.393)	**4.155** (3.026–5.704)	**3.795** (2.930–4.916)
35–44	0.965 (0.563–1.654)	0.891 (0.636–1.247)	**1.947** (1.524–2.487)	**1.356** (1.113–1.653)
45+	1.267 (0.738–2.177)	1.071 (0.764–1.502)	**1.519** (1.164–1.984)	1.152 (0.952–1.394)
Marital status				
Single	ref	ref	ref	ref
Married	1.005 (0.833–1.213)	0.966 (0.845–1.105)	1.108 (0.945–1.301)	0.570 (0.260–1.250)
Widow/divorced	0.626 (0.322–1.214)	0.844 (0.557–1.278)	1.238 (0.767–1.999)	1.080 (0.944–1.236)
Religion				
Christian	ref	ref	ref	ref
Moslem	1.484 (0.494–4.454)	**1.719** (1.214–2.434)	**0.373** (0.266–0.524)	**0.502** (0.398–0.633)
Other	0.554 (0.185–1.660)	0.644 (0.240–1.094)	**0.441** (0.302–0.643)	**0.441** (0.328–0.592)
Education				
Nil	ref	ref	ref	ref
Primary	1.328 (0.930–1.896)	0.880 (0.549–1.412)	**1.547** (1.087–2.697)	1.162 (0.769–1.756)
Secondary	1.162 (0.907–1.489)	0.995 (0.627–1.578)	**2.454** (1.830–3.292)	1.186 (0.763–1.842)
Higher	**1.351** (1.037–1.760)	1.072 (0.617–1.862)	**1.577** (1.181–2.107)	0.631 (0.445–1.546)
Occupation				
Agriculture	ref	ref	ref	ref
Managerial	1.328 (0.945–1.868)	1.265 (0.925–1.730)	2.020 (0.931–4.384)	**0.643** (0.485–0.854)
Sales/service	1.285 (0.822–2.009)	**1.700** (1.164–2.483)	1.289 (0.573–2.897)	**0.702** (0.496–0.996)
Manual	1.222 (0.815–1.832)	**1.788** (1.262–2.534)	1.926 (0.835–4.438)	0.960 (0.821–1.123)
Unemployed	1.181 (0.769–1.815)	1.272 (0.868–1.863)	2.067 (0.948–4.506)	0.571 (0.301–1.086)
Wealth status				
Poorest	ref	ref	ref	ref
Poorer	0.884 (0.464–1.684)	1.132 (0.767–1.673)	1.176 (0.878–1.574)	1.037 (0.855–1.259)
Middle	0.785 (0.224–2.749)	0.876 (0.599–1.280)	1.130 (0.849–1.504)	0.946 (0.773–1.158)
Richer	1.157 (0.376–3.561)	0.727 (0.497–1.063)	**0.728** (0.559–0.949)	0.783 (0.620–1.288)
Richest	**1.624** (1.093–2.413)	0.847 (0.609–1.179)	**0.507** (0.389–0.662)	0.883 (0.617–1.263)
Drinks alcohol				
No	ref	ref	ref	ref
Yes	0.955 (0.791–1.153)	0.942 (0.824–1.076)	**5.828** (4.909–6.920)	**6.388** (5.544–7.361)

N.B. Significant associations are shown in bold. * = adjusted for Age, Marital status, Religion, Education, Occupation, Wealth status. ** = adjusted for Age, Marital status, Religion, Education, Occupation, Wealth status, Drinking alcohol.

## Abbreviations

HIV	Human immunodeficiency virus
LMICs	Low-income countries
NCDs	Non-communicable chronic disease
WHO FCTC	WHO Framework Convention on Tobacco Control
